# Vinculin variant M94I identified in sudden unexplained nocturnal death syndrome decreases cardiac sodium current

**DOI:** 10.1038/srep42953

**Published:** 2017-02-20

**Authors:** Jianding Cheng, John W. Kyle, Brandi Wiedmeyer, Di Lang, Ravi Vaidyanathan, Jonathan C. Makielski

**Affiliations:** 1 Department of Forensic Pathology, Zhongshan School of Medicine, Sun Yat-sen University, Guangzhou 510080, China; 2 Division of Cardiovascular Medicine, Department of Medicine, University of Wisconsin, Madison, WI 53792, USA

## Abstract

Sudden unexplained nocturnal death syndrome (SUNDS) remains an autopsy negative disorder with unclear etiology. Vinculin (VCL) was linked to sudden arrhythmia death in VCL knockout mice prior to the appearance of cardiomyopathy. We hypothesized VCL mutations underlie risk for SUNDS. A rare heterozygous variant VCL-M94I was found in a SUNDS victim who suffered sudden nocturnal tachypnea and lacked pathogenic variants in known arrhythmia-causing genes. VCL was identified to interact with SCN5A *in vitro/vivo*. The VCL-M94I was co-expressed with the cardiac sodium channel in HEK293 cells and also overexpressed in induced pluripotent stem cells derived cardiomyocytes (iPSCs-CM). In HEK293 cells with pH 7.4, VCL-M94I caused ~30% decrease in peak sodium current (*I*_Na_) amplitude compared to WT; under acidotic conditions (pH 7.0) typically found with hypoxia during sleep apnea, M94I resulted in 37% reduction in peak *I*_Na_ compared to WT and the combination of VCL-M94I and pH 7.0 decreased peak *I*_Na_ by ~56% compared to WT at pH 7.4. In iPSCs-CM, similar effects of M94I on reduction of peak *I*_Na_ were observed. This study initially shows both physical and functional interaction between VCL and cardiac sodium channel, and suggests an important role for respiratory acidosis in triggering the fatal arrhythmia underlying SUNDS.

Sudden unexplained nocturnal death syndrome (SUNDS) is prevalent in southeast Asia and is characterized by sudden unexpected death during nocturnal sleep in apparently healthy young males aged 20–40 with structurally normal hearts. It is diagnosed when it remains unexplained after a comprehensive analysis of cause of death, including death scene investigation, gross and microscopic autopsy examination, toxicological screening, and available clinic history review^1^,^2^,^3^. Since its first report in 1917 in the Philippines^4^, diverse hypotheses have been formulated as potential pathogenic mechanisms for SUNDS, including bacterial infection^5^, potassium deficiency^6^, abnormalities of the coronary arteries^7^, nocturnal sleep respiratory disorders^8^, and primary cardiac arrhythmia^2^,^3^. So far, this entity remains inadequately understood with unclear etiology^9^,^10^.

Because of a similar clinic phenotype, and because a Brugada syndrome (BrS) type 1-like ECG appeared in 60% of “survived” SUNDS cases, a coincidence or at least considerable overlap was suggested for the two disorders^2^,^11^. The loss-of-function mutations in the most common BrS-susceptibility gene *SCN5A* were initially linked to SUNDS in 3/10 probands with clinical evidence of SUNDS, and thus SUNDS was proposed to be the phenotypically, genetically, and functionally allelic to BrS^3^. Subsequently, in a larger cohort of 123 SUNDS cases^9^, we identified that the plausible pathogenic rare variants in BrS related cardiac sodium channel encoding genes (*SCN5A, SCN1B*, and *SCN3B*) were present in only 7–13% of Chinese SUNDS victims. Most recently, we identified mutations in the BrS novel susceptible genes *PKP2*^12^ and *SCN10A*^13^ as possible genetic cause of some SUNDS cases. Together these studies suggest that the exploration of new susceptibility genes for the vast majority SUNDS victims are justified and highlight the importance of searching potential pathogenic genes for SUNDS in BrS associated genes.

Inherited cardiomyopathy shares some pathogenic genes (*PKP2, SCN5A, RYR2*) with primary arrhythmia disorders such as BrS^14^,^15^. Mutations in cardiomyopathy susceptibility genes *DSP, DSG2, CASQ2*, and *JUP* were recently genetically linked to BrS^10^,^16^. *Vinculin* (VCL) and its muscle isoform *metavinculin* encode a cytoskeletal protein which connects actin microfilaments to the intercalated disk and membrane costameres in the heart, and was identified as a susceptible gene for dilated cardiomyopathy (DCM) and hypertrophic cardiomyopathy (HCM)^17^,^18^,^19^. Cardiac-myocyte-specific inactivation of the VCL gene in mouse caused high incidence of sudden death in mice younger than 3 months of age despite preservation of normal heart structure and contractile function. The cause of sudden death was observed to be defective myocardial conduction and ventricular tachycardia. The mice that survived developed DCM after 14 weeks and died before 6 months of age^20^.

From these studies, we hypothesized that mutation in VCL may increase the risk for cardiac conduction defect associated ventricular arrhythmia without obvious structural heart disease and account for some cases of SUNDS.

## Methods

### Study Population

44 consecutive SUNDS cases were collected from January 1, 2010 to December 31, 2014 at the National Center for Medicolegal Expertise at Sun Yat-sen University. The inclusion criteria for SUNDS were as previously reported^9^,^10^,^12^,^13^: (1) an apparently healthy individual no younger than 15 years who died of a sudden unexpected death during nocturnal sleep; (2) no history of significant disease; (3) and a negative autopsy, histology, toxicology, and death-scene investigation that resulted in their death being unexplained. Cases with obvious disease or pathological changes to explain the death were excluded. Informed consent was obtained from the legal representatives of the victims. The principles outlined in the Declaration of Helsinki were followed. This study was approved for human research by the ethics committee of Sun Yat-sen University.

### Genetic analysis

The molecular genetic analysis was conducted based on target captured next generation sequencing technology of 80 candidate genes associated with primary arrhythmia and cardiomyopathy as previously reported^10^. Only rare variants resulting in non-synonymous amino acid changes (missense, nonsense, frame-shift insertion/deletions, in-frame insertion/deletions, or splice-errors) and with a minor allele frequency (MAF) <0.01 observed in any ethnic group among population databases including the 1,000 Genome Project (n = 2,504), the National Heart, Lung and Blood Institute Grand Opportunity (NHLBI GO) Exome Sequencing Project (n = 6,503), Exome Aggregation Consortium (ExAC, n = 60,706 all ethnicities, n = 4,327 East Asian) and a local database (n = 2,087,989 of whom were Chinese, with normal phenotype) were considered for further analysis. Rare non-synonymous variants were characterized according to the strict variant interpretation guidelines outlined by the American College of Medical Genetics (ACMG)^21^. All variants reported were confirmed by Sanger sequencing.

### Plasmid constructions of expression vectors

The cDNA for human VCL (mEmerald-Vinculin-N-21) was obtained from Addgen (Plasmid #54304). The VCL construct is a fusion protein with mEmerald attached at the C-terminus of VCL. The VCL-M94I missense mutation was incorporated into VCL-WT using the QuikChange II XL site-directed mutagenesis kit (Stratagene, La Jolla, CA). All clones were sequenced to confirm integrity and to assure the presence of the target mutation and the absence of other substitutions caused by PCR.

### HEK293 cells transfection

The WT or mutant VCL in expression vector was transiently co-transfected with SCN5A (hNav1.5/hH1C1, Genbank accession no. AB158469) subcloned into pcDNA3 at a ratio of 1:4 (0.2 μg: 0.8 μg) into HEK293 cells with FuGENE6 reagent (Roche Diagnostics, Indianapolis, Indiana, USA) in 2 ml media according to manufacturer’s instructions. Transfected cells were identified by mEmerald fluorescence.

### Human iPS-CMs transfection

Human induced pluripotent stem cells derived cardiomyocytes (iPS-CMs) were obtained from Cellular Dynamics International (Madison, WI) and handled according to manufacturer specifications. iPS-CMs were chosen because they have previously been extensively characterized and shown to have a cardiomyocytes phenotype^22^,^23^. The cells were cultured for 11–30 days prior to transfection or cellular electrophysiology experiments. The iPS-CMs were split 24 hours before cellular electrophysiology experiments and plated on 12 mm collagen pre-coated coverslips (BD Biosciences, San Jose, CA). The WT or mutant VCL in mEmerald vector (2.5 μg) was transiently transfected into iPS-CMs with TransIT^®^-LT1 transfection reagent (Mirus Bio LLC, Madison, WI, USA) in 1 ml media according to manufacturer’s instructions.

### Histological study of the heart

To investigate the morphological changes of myocardium, the paraffin-embedded left ventricle sections (6 μm) at the mid-ventricular level from the SUNDS victim and age, gender matched control without structural heart disease were stained with H&E, Masson’s trichrome, and immunofluorescence staining. Immunolabeling was performed as described previously^24^. Sections were stained with commercially available antibodies: Guinea Pig anti-Nav1.5 (polyclonal, Alomone labs, 1:200) and Alexa Fluor 488 Goat anti-Guinea Pig IgG (H + L) (ThermoFisher, Cat# a11073); Mouse anti-VCL (monoclonal, Sigma-Aldrich, 1:200) and Alexa Fluor 568 Goat anti-Mouse IgG (H + L) (ThermoFisher, Cat# a11004). Fluorescent images were acquired using Leica SP5 laser confocal microscope system. SCN5A and VCL antibody were excited at 488 mm and 561 mm respectively, and emission light were filtered by 525 ± 20 mm band-pass filter for SCN5A signals and 605 ± 10 mm band-pass filter for VCL signals. We randomly chose 4 different regions from one section, and two sections from each objective were analyzed. Signal intensity profile of both SCN5A and VCL were calculated along the transverse direction (t-tubule direction), aligned and plotted. Matlab and ImageJ were used for signal and image processing.

### Co-Immunoprecipitation

Adult mouse heart was dissected and homogenized by 10 strikes with a polytron probe in ice-cold RIPA buffer (Roche). Cell extracts were prepared by solubilizing 10^7^ cells in 1 ml of cell lysis buffer (Roche) for 10 min at 4 °C. After brief sonication, the homogenates or cell lysates were transferred to a 1.5 ml tube and rotated for 1 hour at 4 °C, then centrifuged for 10 min at 10,000 g at 4 °C and the supernatant was transferred to a new tube for protein quantification and immunoprecipitation. Immuno-precipitations were performed with Pierce direct IP kit (Thermo Fisher Scientific Inc., #26148), which immobilized 25 μg VCL (Invitrogen, #MA5–11690) or 25 μg SCN5A (Invitrogen, #PA5-34190) antibodies on agarose-resin support directly to improve specificity. Homogenate (1.0 mg/reaction) was mixed with immobilized antibody-agarose resin complex at 4 °C overnight. After washing to remove non-bound (presumably undesired) components of the sample, the antigen is recovered by dissociation from the antibody-agarose resin complex with elution buffer supplied in the kit. The immunoprecipitated samples were analyzed by Western blotting by probing with anti-SCN5A (Sigma, #SAB2107930) or anti-VCL (Sigma, #V4139).

### Electrophysiological measurements

Macroscopic voltage-gated sodium current (*I*_Na_) was measured 24 hours after transfection with the standard whole–cell patch clamp method at room temperature (~22 °C) in both transfected HEK293 cells and iPS-CMs with both normal pH 7.4 and moderate acidosis (extracellular and intracellular pH 7.0). For HEK293 cells, the intracellular solution contained (in mM) CsF 120, CsCl_2_ 20, EGTA 2, NaCl 5, and HEPES 5 and was adjusted to pH 7.4 or 7.0 with CsOH; the extracellular solution contained (in mM) NaCl 140, KCl 4, CaCl_2_ 1.8, MgCl_2_ 0.75 and HEPES 5 and was adjusted to pH 7.4 or 7.0 with NaOH. For iPS-CMs, the bath solution contained (in mM) NaCl 60, CaCl_2_ 1.8, MgCl_2_ 1, CsCl_2_ 105, glucose 10, HEPES 5, and Nifedipine 0.001 and was adjusted to pH 7.4 or 7.0 with CsOH; the pipette solution contained (in mM) NaCl 5, CaCl_2_ 2, CsCl_2_ 135, HEPES 10, EGTA 10, and MgATP 5 and was adjusted to pH 7.4 or 7.0 with CsOH (modified from ref. 22).

Microelectrodes were prepared from borosilicate glass using a Sutter P-87 puller (Sutter Instrument Co, Novato, California, USA). The resistances of microelectrodes ranged from 1.2 to 2.2 MΩ. Voltage clamp data were generated with pClamp software 10.5 and an Axopatch 200B amplifier (Axon Instruments, Foster City, California, USA) with series-resistance compensation. Membrane current data were digitized at 100 kHz, low-pass filtered at 5 kHz, and then normalized to membrane capacitance.

Activation was measured by clamp steps of −120, −110, −100, −90, −80, −70, −60, −50, −40, −30, −20, −10, 0, 10, 30, and 60 mV (the interpulse interval is 2 seconds) from a holding potential of −140 mV. The midpoint of activation was obtained using a Boltzmann function where G_Na_ = [1 + exp (V_1/2_ − V)/k]^−1^, where V_1/2_ and k are the midpoint and slope factor, respectively. G/G_Na_ = *I*_Na_ (norm)/(V − Vrev) where Vrev is the reversal potential and V is the membrane potential. Steady-state inactivation was measured in response to a test depolarization to 0 mV for 24 ms from a holding potential of −140 mV, following a 1 second conditioning pulse from −150 mV to 0 mV in 10 mV increments (the interpulse interval is 2 seconds). The voltage dependent availability from inactivation relationship was determined by fitting the data to the Boltzmann function: *I*_Na_ = *I*_Na-max_ [1 + exp (Vc − V_1/2_)/k]^−1^, where V_1/2_ and k are the midpoint and the slope factor, respectively, and Vc is the membrane potential. Late *I*_Na_ was measured as the mean between 600 and 700 ms after the initiation of the depolarization from −140 mV to −20 mV for 750 ms (the interpulse interval is 2 seconds) after passive leak subtraction as previously described^24^. Time course of recovery from inactivation was elicited using the protocol: holding potential of −140 mV, conditioning pulse to 0 mV for 1 sec, followed by different recovery intervals (from 0.1 to 2000 ms), then a test pulse to 0 mV for 24 ms. The data were analyzed by fitting with a two-exponential (exp) function: normalized *I*_Na_ (*t*) = *A*_f_ [1 − exp(−*t*/τ_f_)] + *A*_S_ [1 − exp(−*t*/τ_S_)], where *t* is time, *A*_f_ and *A*_S_ are fractional amplitudes of fast and slow components, respectively, and τ_f_ and τ_S_ are fast and slow time constant, respectively.

### Statistical analysis

All data points are reported as the mean value and the standard error of the mean (SEM). Determinations of statistical significance were performed using a Student *t*-test for comparisons of two means or using analysis of variance (ANOVA) for comparisons of multiple groups. Statistical significance was determined by a value of *P* < 0.05.

## Results

### Demographics of SUNDS Cohort

The average age of death for 44 unrelated SUNDS victims (1 case was female) was 30.23 ± 8.0 years (range 15–46 years). No clinic record existed for any of these apparently previously healthy SUNDS cases. Comprehensive forensic autopsy examination revealed no significant pathological changes to explain the sudden death. No case was diagnosed with coronary artery disease, cardiomyopathy, viral myocarditis, or congenital heart disease. The hearts of all 44 SUNDS cases were structurally normal. The average heart weight, left ventricle thickness, and right ventricle thickness were 364.78 ± 67.15 g, 1.22 ± 0.18 cm, and 0.32 ± 0.07 cm, respectively.

### VCL Mutational Analysis in SUNDS

**Figure 1:**
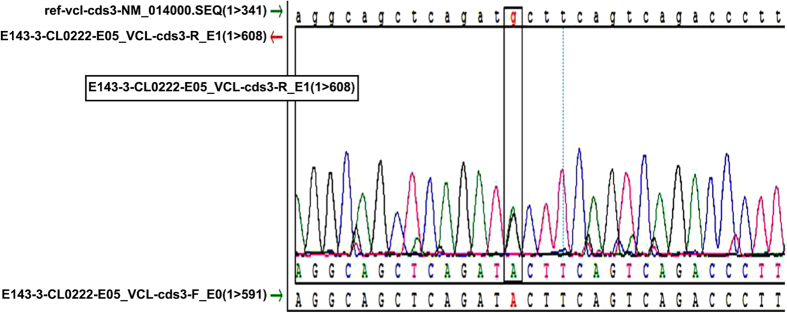
Genotyping of SUNDS victim. The heterozygous VCL variant M94I with a nucleotide change of G-A in the position 282 was identified in a SUNDS victim.

Overall, 22/44 SUNDS cases carried at least one rare non-synonymous variant or Minor Allele Frequency (MAF) <1% among the 80 candidate genes analyzed (data not shown). A rare missense heterozygous variant M94I in VCL (Fig. 1) was identified in a 29-year-old male. No rare variants were identified in known primary arrhythmia causing genes out of 80 genes^10^ screened in this case. M94I was absent in the 1,000 Genome Project, the NHLBI ESP, and local database and with a MAF prevalence of 0.000016 and 0.00023 in ExAC overall population and East Asian, respectively. The case lacked a family history of arrhythmia, sudden cardiac death, and other heart disease. The follow up genetic investigation for the parents of this case did not yield this variant suggesting it may have been a sporadic mutation. M94I was predicted to be malignant by in silico tools SIFT, Polyphen2, and CONDEL. M94I localized to the first head domain (Vh1, amino acids 1–258) which harbors binding sites of talin, α-actin, or α-catenin to VCL^25^,^26^,^27^.

### The histopathological examination of the heart from M94I carrier

The SUNDS victim with M94I was witnessed to have sudden tachypnea and abrupt tic of limbs during nocturnal sleep at approximately 2 am and his roommate immediately transported him to the nearest hospital where he was declared dead. The height, heart weight, left ventricle thickness, and right ventricle thickness were 158 cm, 330 g, 1.0 cm, and 0.3 cm, respectively.

**Figure 2:**
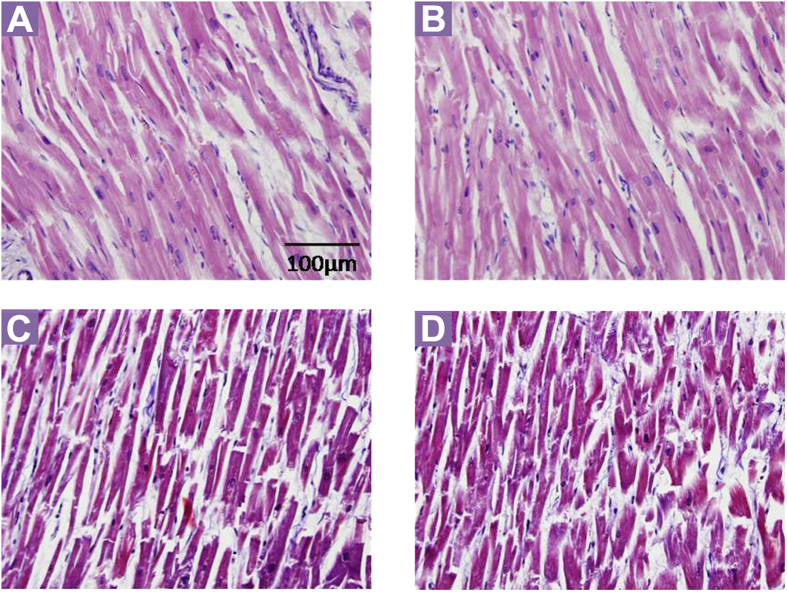
Cardiac histological views. No myocardial hypertrophy, disarray, significant infiltration of inflammatory cells, and substitution of fibrous/adipose tissue in the myocardium were observed in both SUNDS (**A**,**C**) and control (**B**,**D**). (**A**,**B**,**C** and **D**) were stained with H&E, Masson’s trichrome, respectively (all ×400).

**Figure 3:**
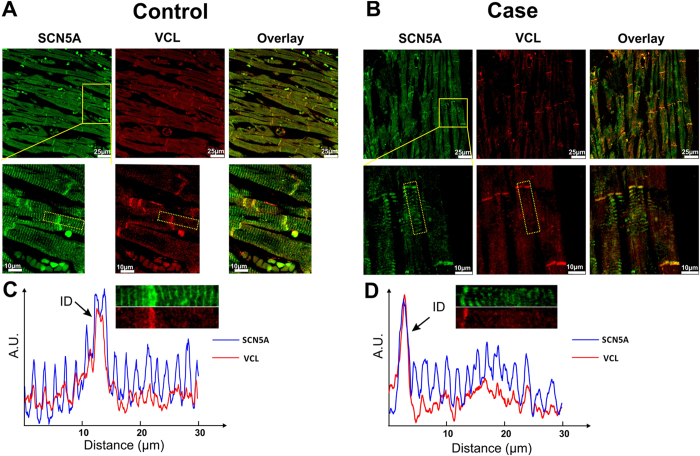
Co-localization of VCL and SCN5A in human ventricular tissue slice. (**A**,**B**) Immunofluorescence staining of SCN5A (green), VCL (red) and overlay channel from control and case (SUNDS patient, VCL–M94I variant carrier) human ventricular tissue slices. The second row shows a higher magnification view of the area outlined in yellow the first row. The yellow color from overlay panel indicates co-localization of VCL and SCN5A. (**C** and **D**) Signal intensity profile of signals from selected areas (outlined with yellow dashed rectangle in second row of (**A** and **B**) were calculated and plotted from control and case slices respectively. Integrity of the fluorescence signals of both SCN5A (blue line) and VCL (red line) along transverse direction was aligned and plotted.

The myocardial sections stained with H&E and Masson’s trichrome from SUNDS case (Fig. 2A,C) showed no myocardial hypertrophy and no significant infiltration of inflammatory cells and fibrous/fatty tissue in myocardium. A tissue control was taken from a 31 year-old male control (died from mechanical asphyxia, no pathogenic rare variant identified in VCL or in known primary arrhythmia causing genes out of 80 genes; Fig. 2B,D). Co-localization of SCN5A and VCL in human left ventricular tissue slices was detected at the intercalated disk (ID) area in an immunofluorescence staining experiment (Fig. 3). The yellow color pixels in the Fig. 3A and B overlay panel suggested that in both control and case images, SCN5A and VCL were co-localized at the ID. Co-localization was quantified by Pearson’s correlation coefficient. The Pearson’s correlation coefficient value is 0.89 and 0.78 (0.5 or more is considered significant) at ID area in control and case slices, respectively, indicating significant co-localization of SCN5A and VCL at ID area. In the signal intensity profile presented in Fig. 3C and D, SCN5A was observed to be expressed at both the ID and t-tubules (blue line) in both control and case groups, whereas VCL (red line) mainly expressed at the ID. No significant difference of expression and distribution patterns of both SCN5A and VCL were observed between control and case groups.

### VCL directly interacts with SCN5A *in vivo* and *in vitro*

**Figure 4:**
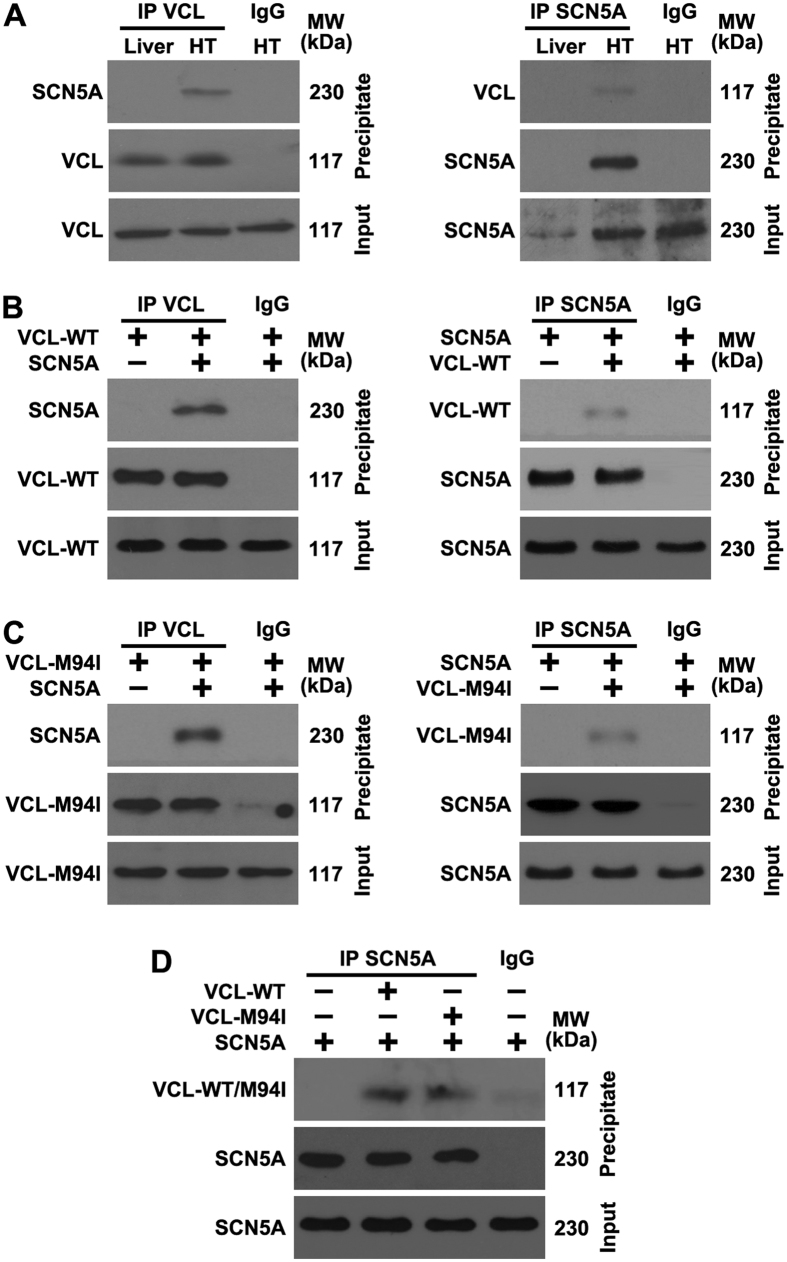
VCL directly interacts with SCN5A. (**A**) Mouse liver and heart (HT) tissue lysates were immunoprecipitated (IP) using VCL or SCN5A antibody and analyzed by Western blotting using the indicated antibodies; (**B**) Wild-type VCL (VCL-WT) and SCN5A were transfected into HEK293 cells for 24 h, cell lysates were IP using VCL or SCN5A antibody and analyzed by Western blotting using the indicated antibodies; (**C**) Mutant VCL (VCL-M94I) and SCN5A were transfected into HEK293 cells for 24 h, cell lysates were IP using VCL or SCN5A antibody and analyzed by Western blotting using the indicated antibodies; (**D**) VCL-WT, VCL-M94I and SCN5A were transfected into HEK293 cells for 24 h, cell lysates were IP using VCL or SCN5A antibody and analyzed by Western blotting using the indicated antibodies. The results are representative of three independent experiments.

To explore the relationship between VCL and SCN5A, we used co-immunoprecipitation experiments to examine if VCL physically interacts with SCN5A. In mouse heart tissues, immunoblot analysis of immunoprecipitated VCL detected the presence of SCN5A, and we also found VCL present following a reciprocal immunoprecipitate using the antibody against SCN5A; due to the negative expression of SCN5A, mouse liver tissue was used as a control (Fig. 4A). The interaction between VCL and SCN5A were also observed in HEK293 cells overexpressing VCL and SCN5A (Fig. 4B), further confirming their direct interaction. Interestingly, we found the interaction between VCL and SCN5A were not affected by the M94I mutation of VCL (Fig. 4C,D). These results suggest that VCL-M94I doesn’t disrupt the physical association of VCL and SCN5A.

### VCL-M94I reduced cardiac sodium current in HEK293 cells

Biophysical characterization of VCL rare variant M94I was performed in HEK293 cells transiently expressing SCN5A and either the wild-type (WT) or the mutant VCL.

**Figure 5:**
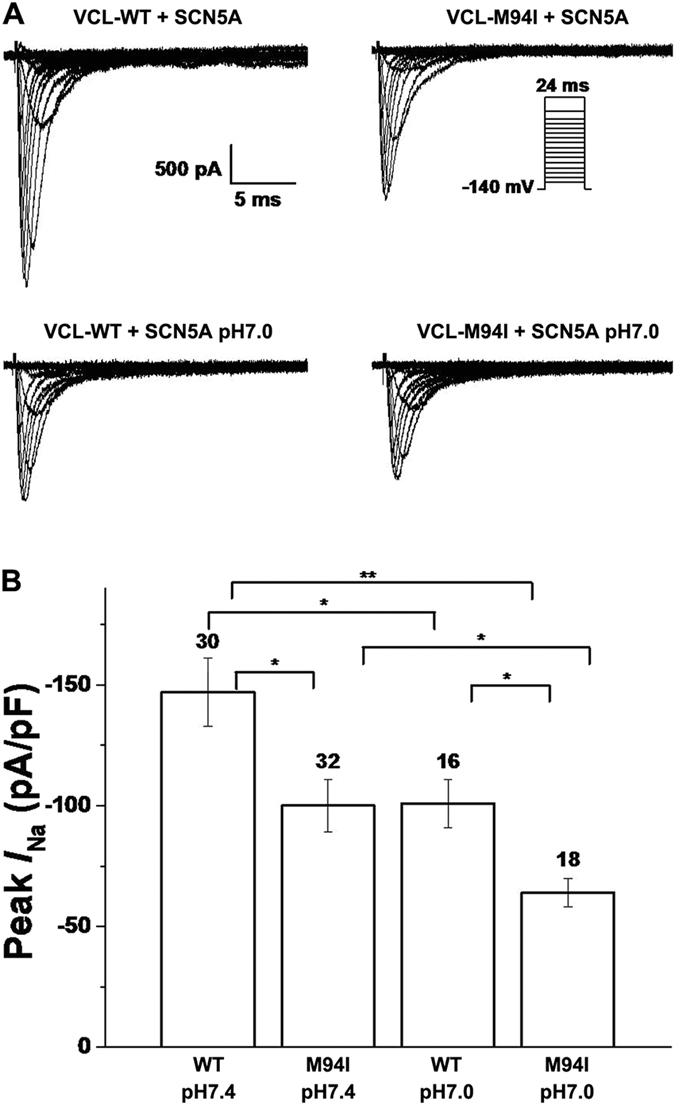
Electrophysiological properties of cardiac sodium channel in HEK293 cells co-expressing SCN5A and either WT or mutant VCL. (**A**) Representative whole-cell current traces showing peak *I*_Na_ under both normal (pH 7.4) and moderate acidosis (pH 7.0) condition in HEK293 cells expressing SCN5A and either WT or mutant VCL. (**B**) Summary data of peak *I*_Na_ densities from every group. The number of tested cells is indicated above the bar. *p < 0.05, **p < 0.01.

**Figure 6:**
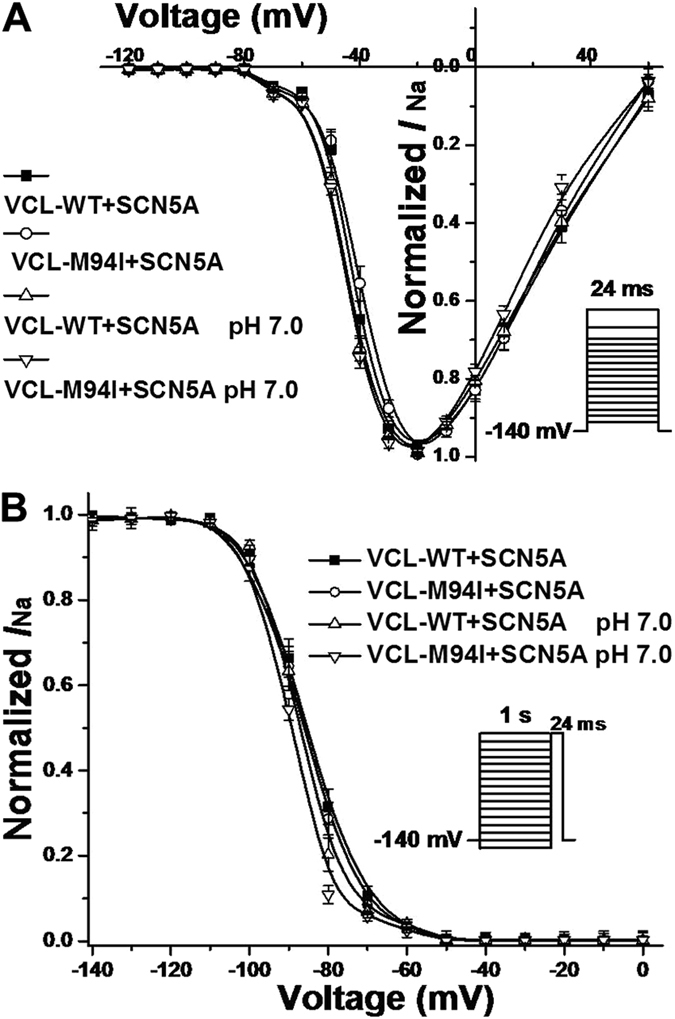
Voltage–dependent gating for SCN5A co-expressed with VCL in HEK293 cells. (**A**) Under normal pH condition, M94I caused a statistically significant depolarizing shift in activation of cardiac sodium channel by 2.7 mV compared to WT. Vrev = 84.8 mV. (**B**) Under pH 7.0, M94I showed a significant repolarizing shift by 4.1 mV in inactivation of cardiac sodium channel compared with WT at pH 7.4.

Under normal pH condition (pH 7.4), M94I caused an approximately 30% decrease in peak *I*_Na_ amplitude compared to WT (Fig. 5A,B; Table 1). The level of late *I*_Na_ was measured as a percentage of peak *I*_Na_ elicited by prolonged depolarization to −20 mV and the results showed the late *I*_Na_ for M94I was comparable to WT (Table 1). The analysis of the kinetic parameters showed that M94I caused a statistically significant depolarizing shift in activation of cardiac sodium channel compared with WT and there was no difference between WT and M94I in inactivation of cardiac sodium channel (Fig. 6A,B; Table 1). For recovery from inactivation of cardiac sodium channel, M94I exhibited slower recovery from inactivation and had significantly bigger both fast and slow time constants (τ_f_, τ_s_) value compared with WT (Table 2). The small positive shift in activation and slower recovery from inactivation of cardiac sodium channel in M94I may account for the 30% reduction in peak *I*_Na_.

Compared with WT at pH 7.4, WT and M94I at pH 7.0 decreased peak *I*_Na_ by ~30% and >50%, respectively (Fig. 5A,B; Table 1). At low pH, M94I caused a significant negative shift by 4.1 mV in inactivation of cardiac sodium channel compared to WT at pH 7.4 (Table 1), and impaired the recovery from inactivation of cardiac sodium channel to slower level compared with M94I at pH 7.4 (Table 2). This negative shift and impaired recovery would be predicted to further decrease of *I*_Na_ at more physiological resting membrane potentials and heart rates.

### Biophysical properties of sodium channels in iPSC-CMs over-expressing VCL-M94I

**Figure 7:**
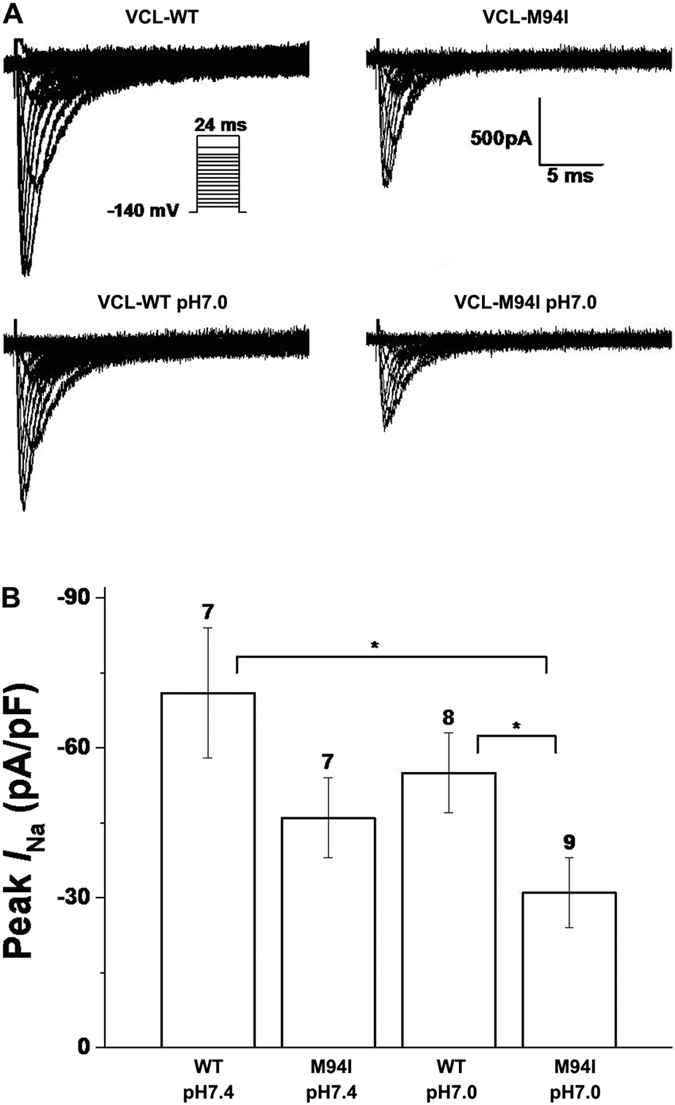
Electrophysiological properties of cardiac sodium channel in iPSC-CMs expressing either WT or mutant VCL. (**A**) Representative whole-cell current traces showing peak *I*_Na_ under both normal (pH 7.4) and moderate acidosis (pH 7.0) condition in iPSCs-CM expressing either WT or mutant VCL. (**B**) Summary data of peak *I*_Na_ densities from every group. The number of tested cells is indicated above the bar. *p < 0.05.

**Figure 8:**
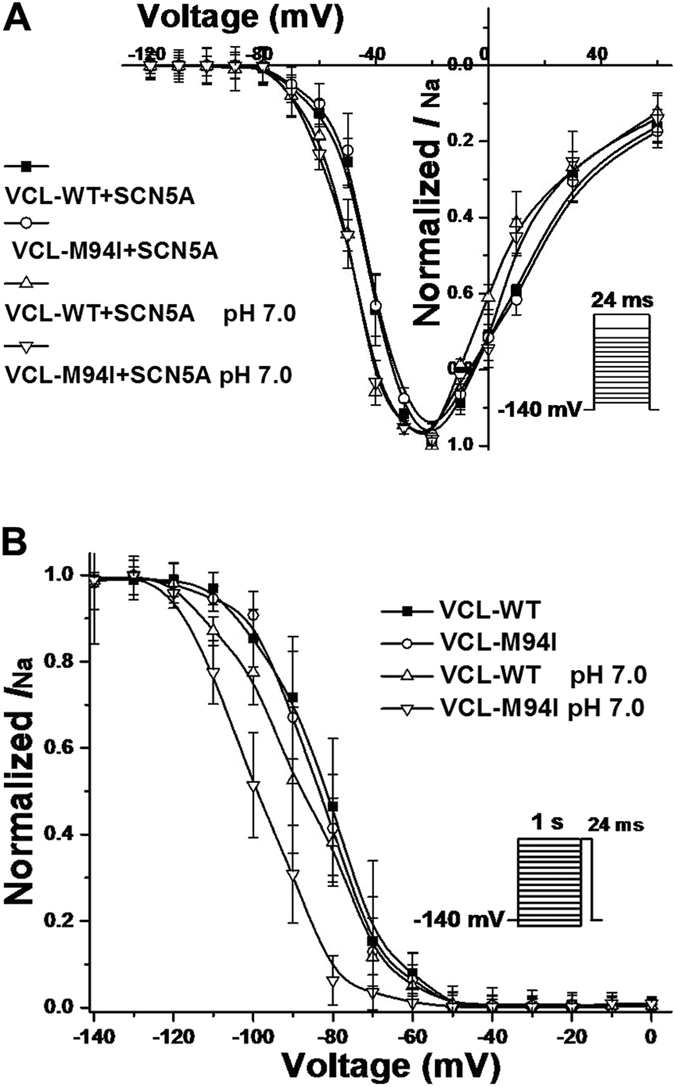
Voltage–dependent gating for cardiac sodium channel in iPSC-CMs expressing with VCL. (**A**) Between each group, no statistically significant difference in activation of cardiac sodium channel was observed. Vrev = 63.2 mV. (**B**) Under pH 7.0, M94I caused a significant repolarizing shift by 15.3 mV in inactivation of cardiac sodium channel compared to WT at pH 7.4.

To observe the effect of mutant VCL M94I on cardiomyocytes, we tested iPSC-CMs over-expressing either WT or mutant VCL. Under baseline pH condition, M94I tended to decrease peak *I*_Na_ compared with WT (Fig. 7A,B; Table 3) without reaching statistical significance (*P* = 0.08). With acidosis at pH 7.0, M94I showed more than 50% reduction in peak *I*_Na_ amplitude compared with WT at pH 7.4 (Fig. 7A,B; Table 3). There was no statistically significant difference in activation (Fig. 8A; Table 3) and recovery from inactivation (data not shown) of cardiac sodium channel between each group. Consistent with the alteration in HEK293 cells, compared to WT at pH 7.4, M94I at pH 7.0 showed a statistically significant negative shift in inactivation of cardiac sodium channel (Fig. 8B; Table 3).

## Discussion

### The functional association of VCL and cardiac sodium channel

As an ubiquitously expressed protein, VCL anchors and assembles the actin cytoskeleton to the cell membrane through integrin- and cadherin-based cellular junctions^25^. In cardiac myocytes, VCL and its muscle splice variant metavinculin are mainly expressed at costameres and intercalated disks (ID) and play a crucial role in maintaining normal cell-cell and cell-matrix junctions as well as cardiac rhythm^20^,^28^,^29^. Although several mutations including L277M, K815R, L954del, and R975W in VCL have been identified to be genetic cause of either DCM or HCM in patients^17^,^18^,^19^,^30^,^31^, the function of VCL in the cardiomyocytes and intact heart is not completely understood. Hemizygous null VCL mice showed normal basal cardiac function and histology but an abnormal ECG and ID^28^. In mice with cardiac-myocyte-specific excision of VCL gene (cVCL-KO), there were two stages of phenotype: one stage with ectopy, complete atrioventricular block, and nonsustained polymorphic ventricular tachycardia, and sudden death within the first 3 months of life, despite preserved systolic cardiac function; the second stage in mice who survived the first stage developed DCM and died of heart failure by around 6 months of age^20^. In both animal models, the gap junctional protein connexin 43 (Cx43) within ID was observed to be abnormally distributed^20^,^28^. Recently, this regulatory effect of VCL on Cx43 was confirmed and attributed to direct interaction between VCL head domain and the third PDZ domain of zonula occludens-1 (ZO-1, a cellular junctions protein) at ID^29^.

Increasing evidence has shown that Cx43 regulates the abundance or localization of SCN5A in the ID subdomain in addition to its canonical functions to form gap junction^32^,^33^,^34^. In this study, we initially identified that VCL interacts directly with SCN5A in both mouse heart and HEK293 cells transiently expressing VCL and SCN5A. Based on these studies, we speculated that dysfunction of VCL may affect sodium channel function. Through biophysical investigation, we have now linked a VCL variant M94I to the loss-of-function of cardiac sodium channel in both HEK293 cells and iPSC-CMs, showing a previously unknown functional interaction between VCL and SCN5A. In the myocardial section from the VCL-M94I carrier, we confirmed the co-localization of VCL and SCN5A at ID, but didn’t find significant difference in distribution of both VCL and SCN5A compared to control. Co-immunoprecipitation also showed that VCL-M94I didn’t affect the physical interaction between VCL and SCN5A. These results suggest the observed functional effect of mutant VCL-M94I on SCN5A does not involve expression and distribution of VCL or SCN5A. The precise mechanism for decreased *I*_Na_ observed (Figs 5 and 7) is unclear. The observed negative shift in inactivation and slower recovery (Tables 1 and 2) of cardiac sodium channel cannot alone account for the observed decrease at the very negative holding potential (−140 mV) and long interpulse interval in those protocols, but it is important to point out that these effects on inactivation and recovery would further decrease *I*_Na_ at normal cardiac resting potentials and heart rates. More detailed biophysical mechanisms for the alteration of sodium channel kinetics and the functional interaction of VCL with SCN5A remain to be identified.

### The role of moderate acidosis in the pathogenesis of SUNDS

Based on forensic epidemiological studies, the abrupt breathing abnormalities during nocturnal sleep (such as sleep apnea, outbursts of tachypnea, screams, strange groans or gaspings, and unusual snore) was one of the most important clinic phenotypes for SUNDS^1^,^2^,^4^,^5^,^6^,^7^. According to a sleep monitoring experiment, nocturnal hypoxia was observed to be the primary abnormality in subjects with family history of SUNDS^35^. The unique Hmong sleep disorders (such as high prevalence of sleep apnea and paralysis) were recently deemed to be relevant to the high prevalence of SUNDS in Hmong males in the USA^8^. All these findings indicated that the nocturnal respiratory disorder may be an important risk factor for the occurrence of SUNDS^36^.

Both extracellular and intracellular pH in cardiac tissue have been found to reach less than pH 6.0 when exposed to no-flow 60-minute ischemia^37^. The arterial blood pH of sudden infant death syndrome (also associated with sleep breathing disorders) was reported to decrease below pH 7.0^38^. Most recently, we showed that moderate acidosis at pH 7.0 significantly exacerbated the loss-of-function of the SCN5A variant identified in Chinese SUNDS victim with sudden nocturnal tachypnea prior to death^39^. In the present study, we hypothesized breathing disorder-related moderate acidosis may trigger the lethal arrhythmia of this SUNDS case with VCL-M94I through effects on the cardiac sodium channel. Compared to VCL-WT under physiological condition, a mild 30% decrease in peak *I*_Na_ caused by VCL-M94I under normal pH was aggravated to over 50% under pH 7.0 condition, and the additive effect of acidosis with the VCL-M94I mutant may cause a sufficiently decreased peak *I*_Na_ to contribute to the sudden arrhythmia death of this case. Our findings provided the first electrophysiological evidence for enhanced effects of acidosis on VCL based cardiac sodium channel function and highlighted the key role of environmental risk factors (such as hypokalemia, medications, acidosis) in the pathogenesis of SUNDS^1^,^4^,^5^,^6^,^7^,^8^,^39^.

### The linkage of cardiomyopathy susceptible gene and primary arrhythmia with apparently normal heart

In both hemizygous null VCL mice and cVCL-KO mice, there was a chronic and progressive pathological change from apparently normal heart to DCM with the increase in age and stress while sudden arrhythmia death could occur at any life stage^20^,^28^. The young VCL-M94I carrier didn’t have any clinic signs, symptoms, or pathological evidence for DCM or HCM and died suddenly and unexpectedly. The biophysical characterization identified VCL-M94I as a conditional arrhythmogenic variant. These studies indicated the cardiomyopathy-susceptibility gene VCL may provide new insights to address the idea that primary arrhythmia disorder is a subtype or early stage of cardiomyopathy^40^,^41^,^42^.

Primary arrhythmia syndromes (such as BrS and idiopathic atrial fibrillation) have been shown to have overlapping clinical phenotypes and share considerable common susceptibility genes (such as *SCN5A, PKP2, ABCC9*, and *RYR2*) with cardiomyopathy^15^,^16^. The rare variants from cardiomyopathy related genes *DSG2, CASQ2, JUP*, and *DSP* were also detected to be plausible genetic cause of some BrS cases^10^,^17^. Most recently, we discovered a 30–40% prevalence of rare variants in 48 cardiomyopathy susceptibility genes in 44 SUNDS victims as well as 17 BrS patients and identified the significant effect of these variants on cardiac morphological alteration and death age^43^.

Besides the overlap of clinic and genetic phenotype, the newest morphological evidence also showed a linkage of cardiomyopathy and primary arrhythmias. Cardiac structural changes such as fibrosis and loss of gap junctions have been identified in BrS cases and were proposed to account for the life-threatening arrhythmia^40^. We also showed increased average heart weight (including enlarged circumference of cardiac valves) in SUNDS victims compared with controls in recent forensic and molecular autopsy investigation on SUNDS^42^. All these findings strongly suggested that there might be an intrinsic association between cardiomyopathy and previously recognized primary arrhythmias with apparently intact heart and the yield of more pathogenic variants in cardiomyopathy related genes such as VCL in BrS and SUNDS would be expected. The absence of structural heart disease in primary arrhythmia syndromes may be more relative than absolute.

### Study Limitations

Although we linked the VCL variant M94I to this SUNDS case by histopathological, molecular, and biophysical evidence, there are limitations in the current study. First, the absence of ECG records is a study limitation inherent to the vast majority of investigations on postmortem cases, especially with SUNDS victims where by the very definition the victims are apparently healthy individuals who die unexpectedly. This has limited a deeper analysis of association between clinical phenotype, genetic findings, and functional data. Secondly, the electrophysiological study was performed by *in vitro* experiments using both HEK293 cells and iPSC-CMs, which do not exactly duplicate the physiological environment *in viv*o. Lastly, since VCL is a cytoskeletal protein with several protein interaction motifs, it may interact with other ion channels, it is possible that this SUNDS-associated VCL variant exerts dysfunctional effects on other cardiac ion channels.

## Conclusions

In conclusion, this study characterized the VCL rare variant M94I identified in a SUNDS victim with a structurally normal heart and provides the first association of a VCL variant to cardiac sodium channel loss-of-function. The distinct aggravation of loss-of-function of sodium channel caused by M94I under moderate acidosis implicates VCL as a new susceptibility gene for SUNDS and highlights that nocturnal sleep respiratory disorders with moderate acidosis may trigger the fatal arrhythmia underlying sudden cardiac death on SUNDS cases with certain inherited genetic defects.

## Additional Information

**How to cite this article:** Cheng, J. *et al*. Vinculin variant M94I identified in sudden unexplained nocturnal death syndrome decreases cardiac sodium current. *Sci. Rep.* **7**, 42953; doi: 10.1038/srep42953 (2017).

**Publisher's note:** Springer Nature remains neutral with regard to jurisdictional claims in published maps and institutional affiliations.

## Figures and Tables

**Table 1:** Biophysical properties of sodium channels in HEK293 cells co-expressing SCN5A and either WT or mutant VCL.

Samples	Peak *I*_Na_		Activation			Inactivation			Late *I*_Na_	
	p*A*/p*F*	n	V_1/2_ (mV)	k	n	V_1/2_ (mV)	k	n	%	n
WT at pH 7.4	−147 ± 14	30	−42.3 ± 0.9	5.0 ± 0.3	23	−85.1 ± 1.3	5.1 ± 0.2	30	0.32 ± 0.06	23
M94I at pH 7.4	−100 ± 11^*^	32	−39.6 ± 0.9*	5.0 ± 0.2	28	−86.6 ± 1.4	5.0 ± 0.2	31	0.37 ± 0.10	20
WT at pH 7.0	−101 ± 10^*^	16	−44.1 ± 0.8	5.7 ± 0.3	21	−87.4 ± 0.9	5.0 ± 0.2	21	0.45 ± 0.23	7
M94I at pH 7.0	−64 ± 6^†‡^	18	−44.6 ± 0.7	5.3 ± 0.3	22	−89.2 ± 1.0^*^	5.0 ± 0.3	21	0.47 ± 0.12	7

*I*_Na_, sodium current; p*A*/p*F* indicates current density; V_1/2_, voltage of half-maximal activation/inactivation; k, slope factor. Values are mean ± SE for n experiments. The late *I*_Na_ level was described as a percentage of peak *I*_Na_. *P < 0.05 versus WT at pH7.4; ^†^*P* < 0.01 versus WT at pH7.4; ^‡^*P* < 0.05 versus M94I at pH7.4 or WT at pH7.0. All parameters were analyzed using one-way ANOVA followed by a Bonferroni test.

**Table 2:** Recovery of sodium channels in HEK293 cells co-expressing SCN5A and either WT or mutant VCL.

Samples	Recovery			
	τ_f_ (ms)	τ_S_ (ms)	*A*_S_	n
WT at pH 7.4	1.82 ± 0.13	29.5 ± 3.3	0.17 ± 0.01	20
M94I at pH 7.4	2.23 ± 0.12^*^	43.9 ± 3.9^†^	0.18 ± 0.01	25
WT at pH 7.0	2.72 ± 0.23^*^	62.5 ± 7.3^†^	0.21 ± 0.01	11
M94I at pH 7.0	3.23 ± 0.15^†‡^	89.7 ± 8.4^†‡^	0.19 ± 0.01	8

*P < 0.05 versus WT at pH7.4; ^†^*P* < 0.01 versus WT at pH7.4; ^‡^*P* < 0.05 versus M94I at pH7.4 or WT at pH7.0. All parameters were analyzed using one-way ANOVA followed by a Bonferroni test.

**Table 3:** Biophysical properties of sodium channels in iPSC-CMs over-expressing either WT or mutant VCL.

Samples	Peak *I*_Na_		Activation			Inactivation		
	p*A*/p*F*	n	V_1/2_ (mV)	k	n	V_1/2_ (mV)	k	n
WT at pH 7.4	−71 ± 13	7	−43.5 ± 3.0	6.2 ± 1.4	13	−80.9 ± 5.5	6.3 ± 0.8	5
M94I at pH 7.4	−46 ± 8	7	−40.8 ± 2.6	6.3 ± 0.9	11	−83.7 ± 4.1	6.8 ± 0.9	5
WT at pH 7.0	−55 ± 8	8	−48.4 ± 1.2	5.0 ± 1.0	7	−84.8 ± 5.5	6.5 ± 1.0	5
M94I at pH 7.0	−31 ± 7^*†^	9	−48.4 ± 3.4	5.0 ± 1.0	5	−96.2 ± 4.6^*^	8.1 ± 0.6	6

*I*_Na_, sodium current; p*A*/p*F* indicates current density; V_1/2_, voltage of half-maximal activation/inactivation; k, slope factor. Values are mean ± SE for n experiments. *P < 0.05 versus WT at pH 7.4; ^†^*P* < 0.05 versus WT at pH 7.0. All parameters were analyzed using one-way ANOVA followed by a Bonferroni test.
